# A model for the detection of pancreatic ductal adenocarcinoma circulating tumor cells

**DOI:** 10.14440/jbm.2018.250

**Published:** 2018-09-05

**Authors:** Matthew S. Alexander, Brianne R. O’Leary, Devon Moose, Juan Du, Michael D. Henry, Joseph J. Cullen

**Affiliations:** 1 Departments of Surgery, University of Iowa College of Medicine, Iowa City, IA 52242, USA; 2 Departments of Molecular Physiology and Biophysics, Pathology and Urology, University of Iowa College of Medicine, Iowa City, IA 52242, USA; 3 Departments of Radiation Oncology, University of Iowa College of Medicine, Iowa City, IA 52242, USA; 4 Holden Comprehensive Cancer Center, Iowa City, IA 52242, USA; 5 Veterans Affairs Medical Center, Iowa City, IA 52246, USA

**Keywords:** pancreatic adenocarcinoma, circulating tumor cells, metastasis, bioluminescent imaging, orthotopic implantation

## Abstract

Metastatic disease is the leading cause of pancreatic ductal adenocarcinoma (PDAC) associated death. PDAC cells invade and enter the bloodstream early, before frank malignancy can be detected. Our objective was to develop an *in vivo* assay enabling the identification and quantification of circulating tumor cells (CTCs) from primary orthotopic PDAC tumors. Human PDAC cells expressing luciferase and green fluorescent protein were orthotopically injected into the pancreas of mice utilizing ultrasound guidance. Bioluminescent imaging was conducted to identify and track tumor growth. CTCs were then isolated and analyzed by flow cytometry to detect GFP-expressing cancer cells. Tumor growth as measured by bioluminescent imaging increased over time. The concentration of CTCs correlated with the strength of bioluminescent imaging signal. In addition, livers bearing macroscopic disease were harvested for further imaging under fluorescence stereomicroscopy and confocal microscopy, which confirmed the presence of metastases. This study represents an orthotopic animal model that reliably detects the presence of CTCs from PDAC. There is a positive correlation between the concentrations of CTCs with overall tumor burden.

## INTRODUCTION

Pancreatic adenocarcinoma (PDAC) is the 3^rd^ leading cause of cancer related deaths in the United States in spite of accounting for only 3.2% of new cancer cases [1]. The lethal nature of this disease can partly be explained by its early proclivity for progression. At diagnosis, 52% of patients already have metastatic disease and an additional 29% have regional lymph node spread, portending very poor 5-year survival rates of 2.7% and 11.5%, respectively [1]. Even patients diagnosed early with stage I disease have 5-year survival rates of only 30%, due to the fact that the majority of patients undergoing surgical resection have either local or distant disease recurrence at a median of 16.2 months following surgery [2-4]. Of individuals who do have disease recurrence, nearly 80% have metastases [3]. The reason for this phenomenon can be attributed to the early metastatic progression of the disease, which can occur before primary tumor is detectable [5]. Some have even proposed the possibility of metastases developing prior to pathologically observable basement membrane invasion and in parallel with primary tumor development [5]. Suffice it to say, PDAC may be a systemic disease and treatment strategies must evolve to better target the systemic disease features.

Circulating tumor cells (CTCs) represent a potential treatment target for systemic disease control. CTCs are tumor cells that detach from primary tumors and enter the circulation with the potential to seed distant tissues and form metastatic disease foci. The concept of CTCs was first described in 1869 [6], but only in the past few decades have technological advances begun to facilitate their detection and characterization. Since then, interest in CTCs has grown tremendously. CTCs have been discovered to have prognostic value and the potential to serve a minimally invasive, real-time “liquid biopsy” method to enhance clinical decision making without the need to expose patients to the risks of potentially dangerous procedures to collect tissue in already frail patients [7,8].

CTCs may originate from primary tumors *via* passive shedding or active migration into blood vessels [9]. In mouse models of pancreatic cancer, active cancer cell intravasation may be facilitated by epithelial-to-mesenchymal transition (EMT) of the cancer cells, which produced a more invasive cellular phenotype [5]. However, using lineage tracing techniques in similar mouse models of pancreatic cancer, it has been demonstrated that cells producing metastatic colonies at distant sites do not necessarily have to undergo EMT [10]. Regardless of their origin, very few CTCs manage to give rise to productive metastatic colonies. This is the likely result of various extrinsic factors that reduce the survival of CTCs during their passage through the circulatory system as well as the intrinsic capacity of individual CTCs to survive and grow in a metastatic end organ such as the liver [11,12]. However, it is reasonable to believe that the number of CTCs is an important factor to consider when it comes to assessing metastatic potential.

Clinically, CTC number has been well known to correlate with overall prognosis in patients with breast, lung, prostate and colorectal cancers [13-16]. More recently, similar correlations have been discovered for pancreatic cancer [17-19]. CTCs have been found in all stages of PDAC but higher CTC numbers are known to correlate with metastatic disease and predict shorter median progression free survival and overall survival [17,18,20-22]. Thus, treatments that produce significant decreases in CTCs may translate to a reduction in metastatic disease potential, and thus improve overall survival. Reproducible and reliable animal models would be invaluable tools to carry out these investigations. In the current study, we demonstrate a novel animal model employing tumor cells modified to facilitate simple identification and quantification in the circulation. Such a model has the potential to design experiments exploring treatments on metastatic behavior of PDAC.

## MATERIALS AND METHODS

### Cell culture

The human PDAC cell line, MIA PaCa-2, was obtained from American Type Culture Collection and passaged routinely for less than 6 months from the time of receiving them. Cells were grown in DMEM high glucose media supplemented with 10% fetal bovine serum and 1% penicillin/streptomycin. Luciferase and GFP expressing MIA PaCa-2 cells were generated using pQClucIN and pMSCV-IRES-GFP plasmids as previously described [23].

### Calibration curve

Luciferase and GFP expressing MIA PaCa-2 cells were added to whole blood in known concentrations (20000 cells/ml, 10000 cells/ml, 5000 cells/ml, 1000 cells/ml, 500 cells/ml, 25 cells/ml and 0 cells/ml). Blood samples underwent immediate red blood cell lysis by addition of ~10 ml of lysis buffer followed by centrifugation at 300 G for 5 min. The resulting supernatant was aspirated and the procedure was repeated. Samples were resuspended in FACS buffer that contained 1 × 10^5^ polystyrene FluoSpheres (Molecular Probes, Eugene OR) per ml. CTCs in each sample were detected and quantified using flow cytometry and a known concentration of microspheres as stated above. FACS buffer containing microspheres that are fluorescent in the scarlet region (645/680) were used to set gates for spheres in forward and side scatter. A sample of blood alone was used to determine where lymphocytes fall in forward and side scatter. A control sample of blood containing a known concentration of tumor cells (1 × 10^6^) was used to identify the population of GFP expressing cells in forward and side scatter. Gates were set for single cells, Hoeschst negative cells and signal intensity. Samples were run on a LSR II (BD Biosciences, *λ*_ex_ = 645 nm and *λ*_em_ = 680 nm) flow cytometer and analysis performed utilizing FlowJO^TM^ software. Results were used to determine circulating tumor cell concentration (CTC/ml) for each sample by the equation [number of CTCs detected/(FluoSpheres detected/100000 FluoSpheres/ml)]. This was correlated to final tumor volume obtained from bioluminescent imaging. The concentration of cells detected correlated with its known concentration by Pearson’s linear regression.

### Orthotopic intrapancreatic injection and bioluminescent image analysis

Female six week old athymic-nu/nu mice were purchased from Envigo and handled in accordance with the University of Iowa Animal Care and Use Committee (IACUC). MIA PaCa-2 cells (4 × 10^5^) expressing luciferase and GFP were injected orthotopically directly into the pancreas of mice using ultrasound guidance as previously described [24]. Cells were suspended in a 1:1 mixture of PBS and Matrigel (Corning, St. Louis, MO) immediately prior to injections to prevent cells from dispersing throughout the abdomen. Tumor formation and growth were monitored weekly through bioluminescent imaging. Mice were given IP injections (200 µl) of a 15 mg/ml solution of VivoGlo^TM^ luciferin (Promega, Madison, WI). Following injection, mice were anesthetized by isoflurane inhalation and imaged using the AMI-1000 (Spectral Instruments Imaging). Total photon flux (photons per second) was quantified (AMIView software) by placing and area of interest around each mouse. Tumor progression was allowed to continue for fifty days post cancer cell injection.

### CTC isolation and analysis

CTCs were isolated from mice following ultrasound guided orthotopic injection. Mice were anesthetized and injected with luciferin prior to cardiac blood draws. Cardiac blood draws were performed using heparinized needles and syringes to obtain roughly 1 ml of circulatory volume. Blood samples underwent immediate red blood cell lysis as described above for the calibration curve experiment. Spearman’s rank correlation was used to assess for significance at the ≤ 0.05 level using GraphPad Prism 7.0 (San Diego, CA).

### *Ex vivo* imaging

Following cardiac draw, the mouse pancreas and liver were removed and *ex vivo* imaging was performed to confirm the presence of a primary pancreatic tumor and identify any hepatic metastatic disease. Organs were kept in the dark and on ice in cold PBS or HBSS prior to and during imaging. Bioluminescence was used to identify the presence of luciferase expression as previously described above. Fluorescent imaging of whole organs for the identification of GFP was performed on an Olympus Fluorescent Stereoscope. Cytoplasmic GFP immunofluorescent staining of pancreas and liver samples fixed in OCT were viewed under 63× magnification. Sections (10 µm) were cut using a MICROM HM 505E, mounted in VectaShield Mounting Medium with DAPI (Vector Laboratories, Burlingame, CA) and viewed with a Zeiss 710 Laser Scanning Confocal Microscope (1 S10 RR025439-01).

### Hepatic tumor cell isolation

Liver specimens with visible metastatic lesions were used to isolate tumor cells to test for the presence of GFP and luciferase expression. Hepatic sections were quickly minced with a scalpel in 5 ml of cold HBSS (with Ca^2+^ and Mg^2+^) on ice. Tissue fragments and buffer were transferred to a conical tube and centrifuged at 450 RCF for 2 min at 4°C. The supernatant was aspirated and 5 ml of 0.5% w/v collagenase made up in HBSS containing Ca^2+^ and Mg^2+^ was added followed by incubation for 5 min at 37°C. After incubation, fragments were resuspended 5–10 times followed by further incubation at 37°C for a total of 30 min. Each resuspension utilized progressively decreasing pipette sizes (25 ml–2 ml). An equal volume of cold HBSS without Ca^2+^ and Mg^2+^ was added followed by centrifugation at 450 RCF for 2 min at 4°C. The supernatant was aspirated and the cell pellet was washed 2 times followed by centrifugation prior to being resuspended as single cells in DMEM high glucose media containing 10% FBS, 1% penicillin/streptomycin and 1% non-essential amino acids. Cells were plated in a T25 tissue culture filter top flask and allowed to attach before being examined for GFP expression.

## RESULTS

### Calibration curve

**Figure 1.**
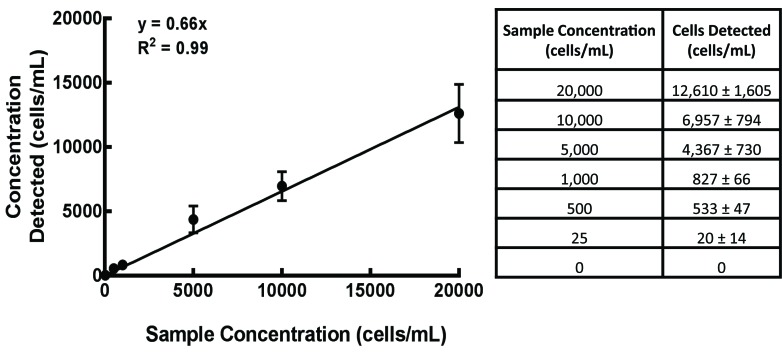
MIA PaCa-2-Luc-GFP calibration curve. Luciferase and GFP expressing MIA PaCa-2 cells were added to whole blood in known concentrations. RBC lysis procedures were carried out and samples were analyzed by flow cytometry for tumor cell quantification. The concentration of cells detected correlated with its known concentration by a Pearson’s linear regression (*y* = 0.66*x*, *r*^2^ = 0.99, Mean ± SEM).

A calibration curve of MIA PaCa-2 cells expressing GFP and luciferase was generated following the addition of known concentrations of cells (range 25–20000 cells/ml) to whole blood and subsequent RBC lysis (**Fig. 1**). Our regression analysis indicates 66% of cells are detected from known concentrations. The correlation of the regression model is significant across the entire range of concentrations tested showing an *r*^2^ value of 0.99.

### Orthotopic intrapancreatic injection and bioluminescent image analysis

**Figure 2.**
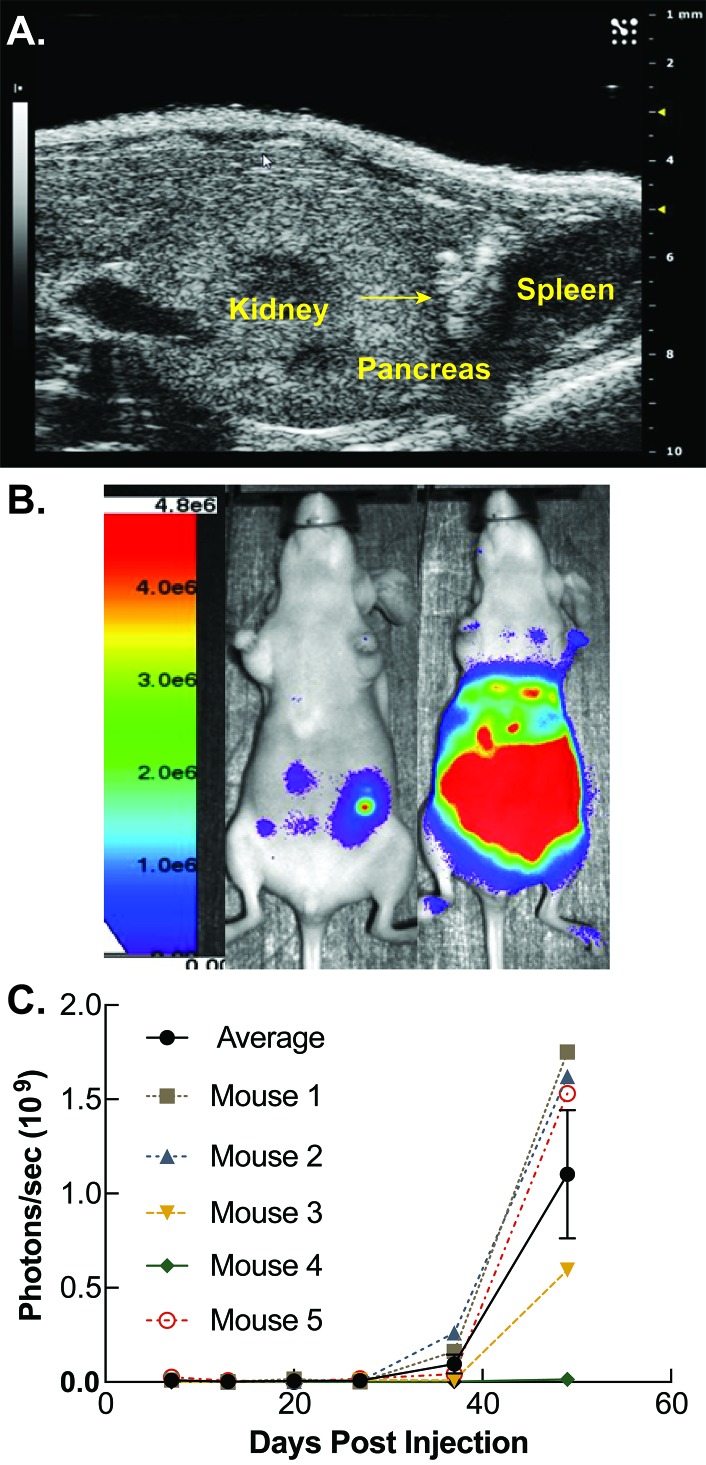
Methods for developing orthotopic circulating tumor cell model. **A.** MIA PaCa-2 pancreatic adenocarcinoma cells (4 × 10^5^) suspended in a 1:1 mixture of PBS and Matrigel were injected into athymic nude mice sedated with isoflurane. Ultrasound guided injections were performed directly into the pancreas medial and inferior to the spleen. The spleen, pancreas, and kidney are labeled. The arrow represents a consolidation of injected cells suspended in Matrigel within the pancreas. **B.** Tumor volumes were measured weekly *via* bioluminescent imaging on an AMI-1000 imager. Mice were imaged 5 min after intraperitoneal injection of 15 mg/ml luciferin. Representative bioluminescent images are shown for days 13 and 50 which display an increase of bioluminescence over time. **C.** Log of photon flux (photons/s) for each mouse and a combined average is shown over the 49 d period following tumor cell injection. Mice displayed a progressive increase of photon flux over time. Tumor growth over time increased by an average of 1.1 × 10^9^ ± 0.7 × 10^9^ photons/s. A region of interest was placed around each mouse and total photon flux was quantified utilizing the AMIView software. Data reported as means ± SEM of total photon flux (photons per second).

Ultrasound guided injections were performed directly into the pancreas medial and inferior to the spleen. This “V”-shaped pocket between the kidney and spleen was visualized by ultrasound as demonstrated in **Figure 2A.** Tumor cells were injected into the lower apex of this region and can be directly visualized following injection as a region of hyperechogenicity as indicated by the arrow (**Fig. 2A**). Weekly bioluminescent expression was quantified for each mouse and tracked over time. A representative color scaled image indicating photons/s superimposed over a photograph from mice on days 13 and 49 are shown in **Figure 2B.** Progression of the average tumor growth curve for each of the five mice injected with tumor is shown in **Figure 2C.** Four out of five mice injected with PDAC showed progressive increases in photon flux compared to background throughout the duration of the experiment. The combined tumor growth increased over time with the final average being 1.1 × 10^9^ ± 0.7 × 10^9^ photons/s (**Fig. 2C**).

### Confirmation of GFP expression

**Figure 3.**
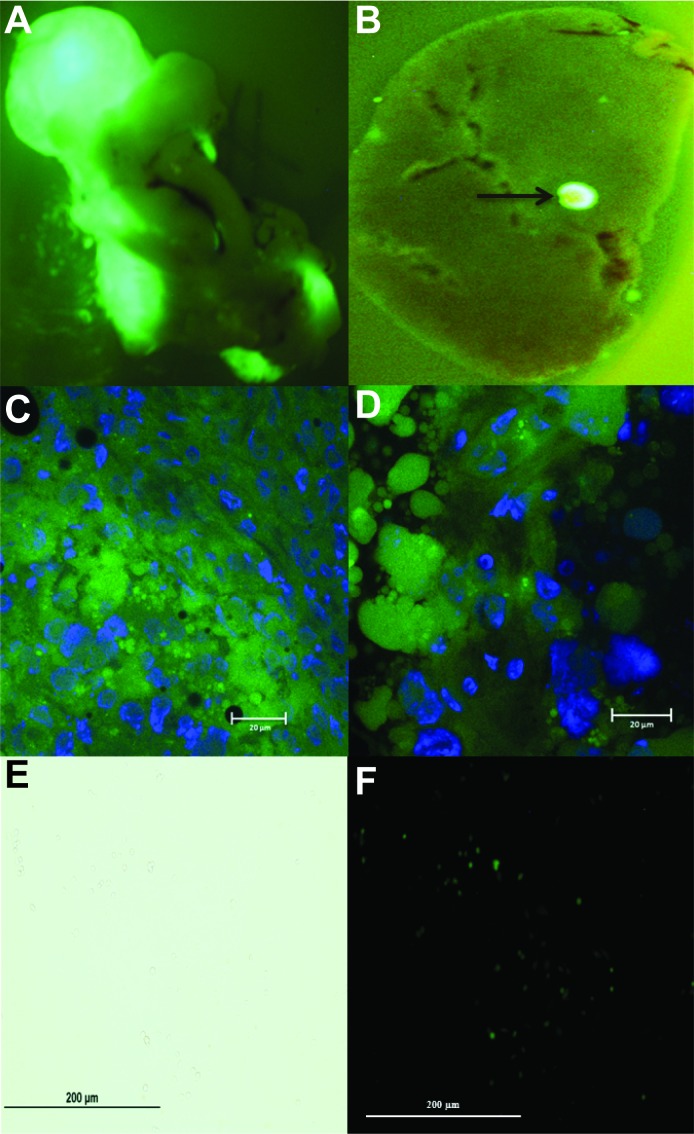
*Ex vivo* pancreatic and liver GFP fluorescent imaging for primary and metastatic tumor identification. **A.** Immediately following cardiac draw and mouse sacrifice, the pancreas and liver were removed for ex vivo imaging under an Olympus Fluorescent Stereoscope to confirm primary pancreatic tumor from GFP expressing MIA PaCa-2 cells. **B.** Liver metastasis from GFP expressing MIA PaCa-2 cells. Arrow corresponds to liver metastasis. **C.** GFP immunofluorescent staining of primary pancreatic tumor. **D.** GFP immunofluorescent staining of liver metastasis. Specimens were fixed in OCT for immunofluorescent staining, cut into 10 µm section using a MICROM HM 505E, mounted in VectaShield Mounting Medium with DAPI and viewed with a Zeiss 710 Laser Scanning Confocal Microscope. Green areas represent positive GFP staining within the cell cytoplasm in both pancreatic and liver specimens. Blue areas represent cell nuclei. **E.** Bright field. **F.** GFP expression. Liver specimens with visible metastatic lesions were used to isolate single tumor cells to test for the presence of GFP expression. Hepatic sections were quickly minced with a scalpel in cold HBSS and centrifuged before the addition of 0.5% w/v collagenase. Cells were plated and allowed to attach before being examined for GFP expression at 20× magnification.

Orthotopic primary pancreatic tumors were confirmed by *ex vivo* GFP microscopy in the explanted whole pancreas (representative image, **Fig. 3A**). In addition, two mice (# 1 & 5) were found to have small foci of metastatic disease in the liver as confirmed by *ex vivo* GFP microscopy (representative image, **Fig. 3B**). GFP immunofluorescence was also confirmed in the cell cytoplasm of primary pancreatic tumor and liver metastases fixed in OCT, sectioned and imaged under magnification (representative images, **Fig. 3C** and **3D**). Single cell isolation from hepatic tumor metastases was performed and confirmed by GFP microscopy (representative image, **Fig. 3E** and **3F**).

### Circulating tumor cell identification and quantification

**Figure 4.**
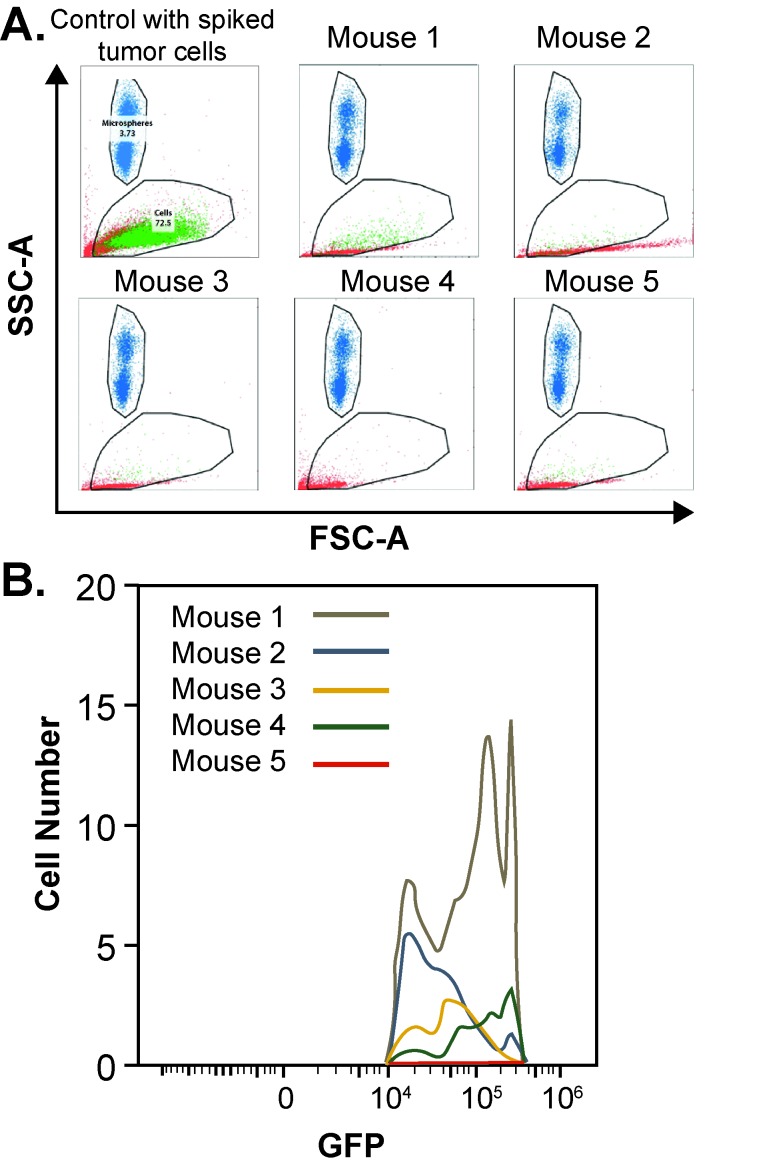
Flow cytometry detection of circulating tumor cells. **A.** Lysed preparation of red blood cells made up in FACS buffer containing polystyrene FluoSpheres was used for quantification. Samples demonstrate gating of microspheres, which are shown in blue, while gating of GFP expressing circulating tumor cells are shown in green, and all other cells which are present but not quantified, are shown in red. **B.** Histogram indicating the total number of CTCs detected in circulation for each mouse.

**Figure 5.**
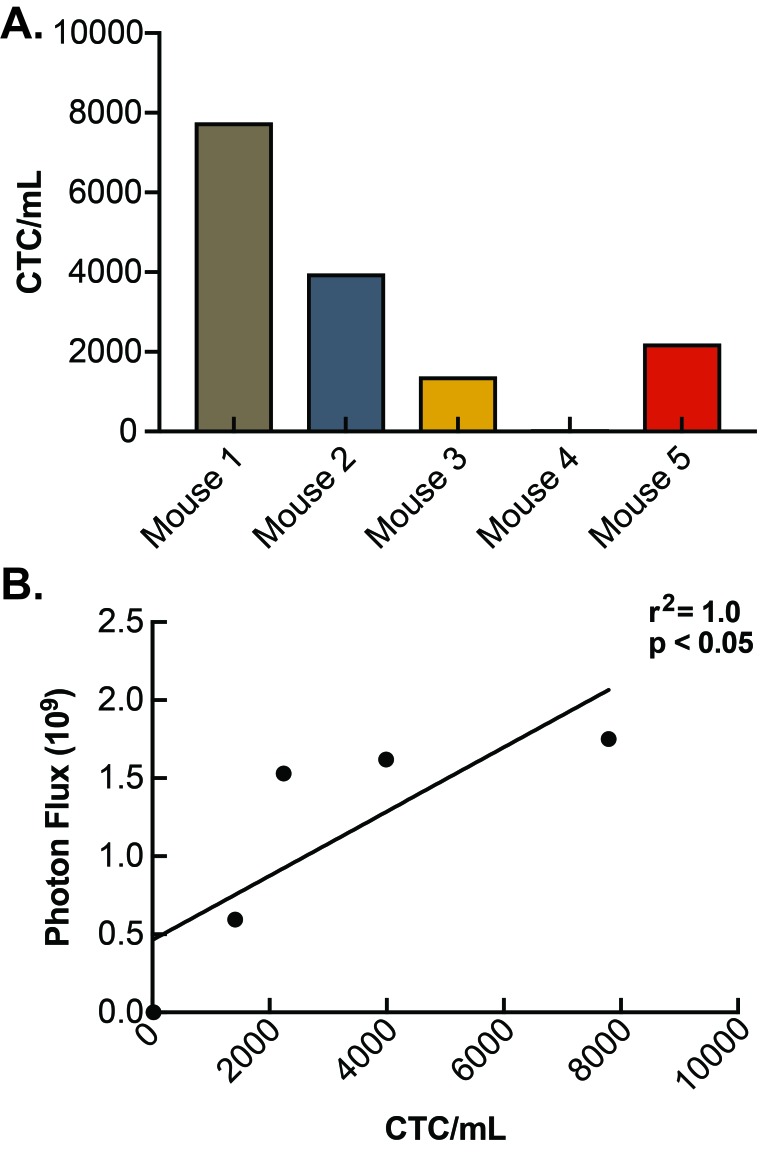
Determination of circulating tumor cell concentration. **A.** CTC concentration (CTCs/ml) was determined for each mouse. Mouse #4 contained the lowest concentration (21 CTCs/ml) and mouse #1 contained the highest concentration (7791 CTCs/ml). **B.** Serum CTC concentration was plotted against overall final tumor burden and a Spearman’s rank correlation for linear regression confirmed correlation and was found to be significant (*r*^2^ = 1.0; *P* < 0.05).

Serum samples processed through RBC lysis were evaluated by flow cytometry and sorted for GFP expression to indicate tumor cells shed into circulation (**Fig. 4A**). A known concentration of microspheres allowed the exact volume passing through the flow cytometer to be determined and facilitated an accurate quantification of CTCs concentration. All of the mice injected with PDACs displayed detectable levels of CTCs with GFP expression and were compared to a known control for quantification. A histogram indicating the total number of CTCs detected by flow cytometry for each mouse is shown in **Figure 4B.** CTCs concentration ranged from 21 CTCs/ml to 7791 CTCs/ml (**Fig. 5A**) with a mean concentration of CTCs of 3092. The concentration of CTCs for each mouse was then plotted against overall final tumor burden and a linear regression model was produced (**Fig. 5B**) demonstrating a positive correlation (*r*^2^ = 1.0; *P* < 0.05).

## DISCUSSION

Cancer treatment in general has improved immensely over the last quarter century; however, treatment breakthroughs for PDAC have been elusive. PDAC is highly aggressive and often metastasizes well before symptoms develop precluding surgical options for cure [1]. Even localized disease commonly recurs in distant sites following surgical resection and adjuvant chemotherapy [25], perhaps indicating both the high frequency of undetectable metastatic disease in these patients and the high level of resistance this disease has to current systemic chemotherapies. Thus, PDAC is a highly aggressive systemic disease, but the systemic dimension is poorly understood. A principle challenge in accumulating reliable systemic disease data has been a lack of reliable methodology to measure systemic disease and metastatic potential. Circulating tumor cells are widely thought to be the link between primary tumor and metastatic disease [26]. The purpose of this study was to develop a method for detecting and quantifying circulating tumor cells in mice inoculated with primary human PDAC as a tool to study the systemic dimension of this aggressive disease.

Our results show that PDACs from whole blood can be reliably detected by flow cytometry and may be quantified over a wide range of concentrations making it possible to measure levels of circulating tumor cells *in vivo*. The present study provides a novel animal model to detect and quantify PDAC CTCs in orthotopic xenografts. Over the seven week study period, pancreatic tumors grew appreciably. The correct anatomic placement of the tumor is critical to the design of the model and has two key considerations. First, orthotopic placement enables primary tumor cells to enter the hepatic circulation as they would in human subjects via the portal system, as opposed to the systemic circulation which is the route of entry for heterotopic xenograft models. This theoretically preserves the metastatic circulatory pathway under study and provides a high fidelity method to study tumor cells entering circulation. Indeed, foci of metastatic disease were discovered in the examined liver tissue and confirmed by GFP expression under stereomicroscopy and *ex vivo* bioluminescent imaging. Secondly, CTCs collected by cardiac draw from orthotopic tumors are filtered through the liver when compared to flank tumors and may reduce the number of detectable CTCs. However, when applied consistently, this method can be used as an objective way to measure how various treatments affect the circulating tumor volume and whether those treatments translate to alterations in metastatic potential.

Interestingly, this data also demonstrated a correlation between the concentration of CTCs and the size of the solid tumor burden, as indicated by bioluminescent signal strength. This may suggest that larger PDAC tumors are likely to shed more metastatic cells into circulation where there is opportunity to seed distant tissues as metastatic disease foci. This finding is consistent with correlations already known to be true that larger cancers are more likely to spread [27].

There are certain limitations to this study. Notably, it is impossible to distinguish primary tumors from metastatic lesions with abdominal bioluminescent images alone due to light scatter and refraction within the abdomen. However, the bioluminescent intensity does provide a good measure for total solid tumor burden. In our experience, the most reliable way to determine the presence of metastatic disease is by post mortem pathologic examination in combination with *ex vivo* bioluminescent imaging. Additionally, confirmation of primary tumor in the pancreatic parenchyma is limited to evidence supplied by immediate visualization on ultrasound imaging and post mortem pathologic examination demonstrating tumor in the pancreas.

Circulating tumor cell detection is not currently part of the standard clinical workup for patients with suspected or confirmed pancreatic cancer. However, there have been recent improvements in the accuracy of human PDAC CTC detection methods. As a diagnostic tool, one study has reported a CTC detection sensitivity and specificity of 75% and 96%, respectively with an area under the receiver operating characteristic curve (AUROC) of 0.867 [18]. There is significant prognostic value as well. Higher CTC numbers (< 3 CTCs/ml) have been shown to trend toward lower median overall survival (11.5 months *vs*. 20 months) [17] and cell surface phenotype expression (epithelial *vs*. mesenchymal) also appears to predict disease recurrence and survival [19]. Considering the progress thus far, it is conceivable that CTC detection and enumeration may assist with diagnosis, staging, prognostication, and treatment response monitoring for patients with PDAC.

In summary, our analysis describes a dependable animal model for studying PDAC metastases. Genetically modified orthotopic PDAC tumor xenografts can produce detectable CTCs and the concentration is dependent on the amount of tumor burden. This is the first study detecting CTCs in mice utilizing human orthotopic PDAC xenografts which carries significant potential for future study as a reliable tool to identify treatments with a high capacity for decreasing the number of tumor cells in circulation and potentially generating patient survival advantages.
